# Supplementary material for: Prognostic relevance of an epigenetic biomarker panel in sentinel lymph nodes from colon cancer patients
Source: Clin Epigenetics. 2017 Sep 5;9:97. doi: 10.1186/s13148-017-0397-4 (PMC5584052; doi:10.1186/s13148-017-0397-4)
Supplement: Additional file 1: Figure S1. — Venn diagram illustrating the overlap between HES-positive and DNA methylation biomarker-positive lymph nodes. (A) Individual lymph node level. (B) Patient level. Abbreviation: HES, hematoxylin-erythrosin-safranin. (PDF 146 kb) [file 13148_2017_397_MOESM1_ESM.pdf]

Additional file 1: Figure S1

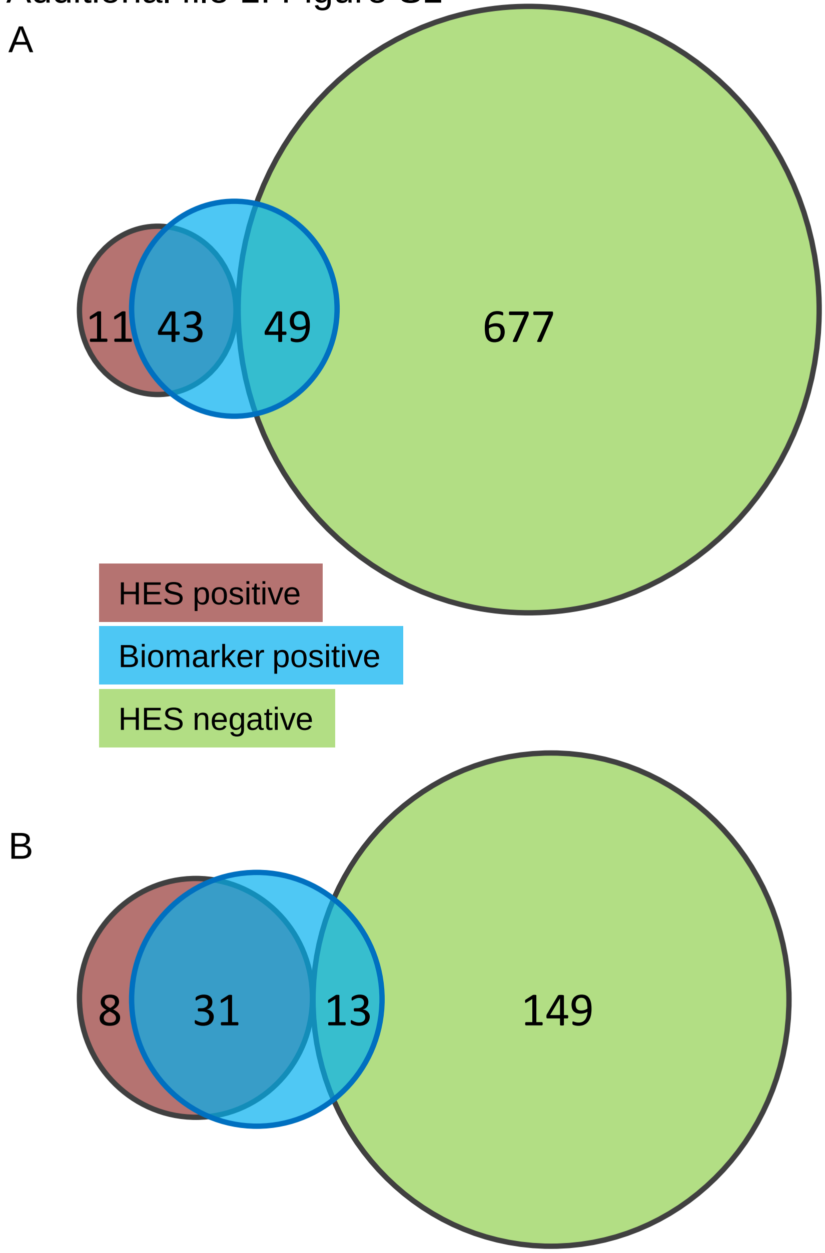

Figure legend: **Venn diagram illustrating the overlap between HES positive and DNA methylation biomarker positive lymph nodes.** A) Individual lymph node level. B) Patient level. Abbreviations: HES, hematoxylin-erythrosin-safranine.
